# Comparative study of inverted internal limiting membrane (ILM) flap and ILM peeling technique in large macular holes: a randomized-control trial

**DOI:** 10.1186/s12886-018-0826-y

**Published:** 2018-07-20

**Authors:** Naresh Babu Kannan, Piyush Kohli, Haemoglobin Parida, O. O. Adenuga, Kim Ramasamy

**Affiliations:** Department of Vitreo-retinal services, Aravind Eye Hospital and Post graduate Institute of Ophthalmology, Madurai, Tamil Nadu India

**Keywords:** 600 μm, Inverted ILM flap, Large macular hole, Muller cells, Type 1 closure

## Abstract

**Background:**

The anatomical success rate of macular hole surgery ranges around 93–98%. However, the prognosis of large macular holes is generally poor. The study was conducted to compare the anatomical and visual outcomes of Internal Limiting Membrane (ILM) peeling vis-a-vis inverted ILM flap for the treatment of idiopathic large Full-Thickness Macular Holes (FTMH).

**Methods:**

This was a prospective randomized control trial. The study included patients with idiopathic FTMH, with a minimum diameter ranging from 600 to 1500 μm. The patients were randomized into Group A (ILM peeling) and Group B (inverted ILM flap). The main outcome measures were anatomical and visual outcome at the end of 6 months. Anatomical success was defined as flattening of macular hole with resolution of the subretinal cuff of fluid and neurosensory retina completely covering the fovea.

**Results:**

There were 30 patients in each group. The mean minimum diameters in Group A and B were 759.97 ± 85.01 μm and 803.33 ± 120.65 μm respectively (*p* = 0.113). The mean base diameter in group A and B was 1304.50 ± 191.59 μm and 1395.17 ± 240.56 μm respectively (*p* = 0.112). The anatomical success rates achieved in Group A and B were 70.0 and 90.0% respectively (*p* = 0.125). The mean best-corrected visual acuity (BCVA) after 6 months was logMAR 0.65 ± 0.25 (Snellen equivalent, 20/89) in Group A and logMAR 0.53 ± 0.20 (Snellen equivalent, 20/68) in Group B (*p* = 0.060). The mean improvement in BCVA was 1.4 lines and 2.1 lines in groups A and B respectively (*p* = 0.353). BCVA≥20/60 was achieved by 13.3 and 20.0% in group A and B respectively (*p* = 0.766).

**Conclusion:**

The anatomical and functional outcome of Inverted ILM flap technique in large FTMH is statistically similar to that seen in conventional ILM peeling.

**Trial registration:**

Clinical Trials Registry – India (Indian Medical Research) CTRI/2017/11/010474.

**Electronic supplementary material:**

The online version of this article (10.1186/s12886-018-0826-y) contains supplementary material, which is available to authorized users.

## Background

Vitrectomy is the gold standard for the treatment of macular holes. The anatomical success rate of macular hole surgery is 93–98% [1–4]. However, the anatomical success rate of large macular holes is as low as 40 to 80% [5–10]. Poor prognosis demoralizes most surgeons from operating upon such patients [11–13].

Michalewska et al., first described a novel technique of inverted internal limiting membrane (ILM) flap for the treatment of large macular holes [14]. They found that their technique achieved better anatomical and visual outcomes compared to conventional ILM peeling (ILMP). In last couple of years, a number of studies have suggested that inverted ILM flap technique (IFT) may be better for the treatment of large macular holes [14–24]. A systemic review and single-arm meta-analysis showed that the anatomical closure and visual improvement rates after IFT for FTMH with minimum diameter (MD) > 400 μm were 95 and 75% respectively [19]. But most of these studies were either retrospective in nature or lacked a control arm and included macular hole with MD < 600 μm.

We performed a randomized control trial to compare the anatomical and visual outcome of inverted ILM flap technique (IFT) vis-à-vis conventional ILM peeling (ILMP) in idiopathic large macular holes with MD > 600 μm.

## Methods

This was a prospective randomized control study done at Aravind Eye Hospital, Madurai, India, after obtaining approval from Institutional Review Board (IRB) of Aravind Medical Research Foundation (Registration No. ECR/182/Inst/TN/2013 dated 20.04.2013). This study adheres to the tenets of Declaration of Helsinki. The nature and aim of the study was explained to the patients, and a written consent for participation was taken from each patient before the surgery. Patients with idiopathic full thickness macular hole (FTMH) with a MD > 600 μm were included. Patients with MD > 1500 μm, traumatic macular holes, myopic macular holes, presence of co-existing ocular pathologies affecting vision and patients refusing randomization were excluded from the study.

The presenting best-corrected visual acuity (BCVA) and intraocular pressure (IOP) were recorded. The Snellen visual acuity was converted into a logarithm of the minimum angle of resolution i.e. logMAR for statistical analysis. FTMH parameters and indices were gauged with Heidelberg Spectralis Spectral-Domain Optical Coherence Tomography (SD-OCT) (Heidelberg Engineering, Inc., Heidelberg, Germany) using high definition 5-line raster scans and 3-dimensional 512 × 128 macular cube scans passing through the fovea, before and after the surgery [6, 7, 9]. All the surgeries in both the groups were performed by a single surgeon (Dr NB) with more than 15 years of post-fellowship experience of performing high volume vitreoretinal surgeries. All the patients were randomized into two groups. System-generated random number were used to recruit the patients into two groups. The patients in group A underwent 25G Pars Plana Vitrectomy (PPV) with ILMP while the patients in group B underwent 25G PPV with IFT.

### Surgical technique

In both groups, phacoemulsification with implantation of intraocular lens was followed by core vitrectomy and induction of posterior vitreous detachment. ILM was then stained with 0.05% solution of Heavy Brilliant Blue G dye (HBBG), prepared by mixing Brilliant Blue G dye (Ocublue plus, Aurolab, India) with 10% dextrose in 1:2 proportions. HBBG was injected slowly under balance salt solution (BSS) [25]. The stained ILM was pinched with a 25G end gripping forceps (Grieshaber Asymmetrical Forceps, DSP, Alcon, Fort Worth, Texas, USA) and peeled off in a circular fashion for approximately 2-disc diameters around the hole.

In the ILM peeling group, the ILM was discarded. In the inverted ILM flap group, the margins of the ILM were left attached to the edges of the hole. The margins were later trimmed with the vitrectomy cutter. Only adequate amount of ILM required to tuck into the hole was retained. Fluid-air exchange (FAE) was done and the ILM flap was tucked into hole with Tano diamond-dusted membrane scraper (DDMS; Synergetics, Inc., O’Fallon, MO, USA).

In both groups, FAE was done multiple times to ensure complete fluid removal. The superonasal and superotemporal cannulae were then removed and the conjunctiva was repositioned to cover the sclerotomy sites. Two mL pure SF_6_ was injected with a 30-gauge needle, while the air-infusion line was used for venting. After the syringe was flushed, the infusion line was clamped and the digital tension of the globe was assessed. The infusion cannula was then removed and the inferotemporal sclerotomy sealed [26]. Post-operative prone positioning was recommended for first 48 h.

### Post-operative evaluation

Post-operative visits were scheduled at day1, 2 weeks, 1 month and 6 months. Frequent follow-ups were scheduled, in case of any complication. At each follow-up visit BCVA, IOP and SD-OCT were recorded. The main outcome measures were anatomical and visual outcome at the end of 6 months. Anatomical closure was defined as the flattening of the hole with resolution of subretinal cuff of fluid. Anatomical success was defined as Type 1 anatomical closure i.e. flattening of macular hole with resolution of subretinal cuff of fluid and neurosensory retina (NSR) completely covering the fovea [27]. Type 2 anatomical closure, i.e. when the whole rim of the NSR around the macular hole was attached to the underlying retinal pigment epithelium (RPE) but NSR was absent above the fovea, was also considered anatomical failure.

### Statistical analysis

Statistical analysis was performed by using statistical software STATA 14.1, (Texas, USA). Continuous variables were expressed as mean (±standard deviation) or median (range) and categorical variables were expressed as percentages. Chi-square test/ Fisher’s exact test was used to assess the association of categorical variables. Student’s t-test/ Mann-Whitney U test was used to find out the significant difference of continuous variables between the two study groups. Wilcoxon sign rank test was used to find out the difference between pre- and post-operative visual acuity. *P*-value less than 0.05 considered as statistically significant.

### Sample size calculation

The type 1 closure rate obtained by Michalewska et al., i.e. 69% in ILMP group and 96% in IFT group, was used as reference [14]. By keeping the power of the study as 80% and the confidence interval as 95%, a sample of 60 subjects (30 in each arm) was calculated. The following formula was used:

$$ {\mathrm{H}}_{\mathrm{o}}:{\mathrm{P}}_1={\mathrm{P}}_2;\kern0.5em {\mathrm{H}}_{\mathrm{a}}:{\mathrm{P}}_1\ne {\mathrm{P}}_2 $$

$$ \mathrm{n}\kern0.5em =\kern0.5em \frac{{\left\{{Z}_{1-\frac{a}{2}}\;\sqrt{2\;\overline{\mathrm{P}}\;\left(1\hbox{-} \overline{\mathrm{P}}\right)}+{\mathrm{Z}}_{1\hbox{-} \beta}\;\sqrt{{\mathrm{P}}_1\;\left(1-{\mathrm{P}}_1\right)+{\mathrm{P}}_2\left(1-{\mathrm{P}}_2\right)}\right\}}^2}{{\left({\mathrm{P}}_1-{\mathrm{P}}_2\right)}^2} $$

Where,

$$ \overline{\mathrm{P}}=\frac{{\mathrm{P}}_1+{\mathrm{P}}_2}{2} $$

$$ {\displaystyle \begin{array}{ll}{\mathrm{P}}_1& :\mathrm{Proportion}\kern0.17em \mathrm{in}\kern0.17em \mathrm{the}\kern0.17em \mathrm{first}\kern0.17em \mathrm{group}\\ {}{\mathrm{P}}_2& :\mathrm{Proportion}\kern0.17em \mathrm{in}\kern0.17em \mathrm{the}\kern0.17em \mathrm{second}\kern0.17em \mathrm{group}\\ {}\upalpha & :\mathrm{Significance}\kern0.17em \mathrm{level}\\ {}1-\upbeta & :\mathrm{Power}\end{array}} $$

## Results

The study included 30 patients in each of the two groups. The mean MD in group A (ILMP) and group B (IFT) was 759.97 ± 85.01 μm and 803.33 ± 120.65 μm respectively (*p* = 0.113). The mean base diameter in group A and B was 1304.50 ± 191.59 μm and 1395.17 ± 240.56 μm respectively (*p* = 0.112). The mean BCVA in group A and B was logMAR 0.79 ± 0.24 (Snellen equivalent 20/123) and logMAR 0.75 ± 0.22 (Snellen equivalent 20/112) respectively (*p* = 0.471) (Table 1).

**Table 1:** The baseline characteristics of the two groups

	Group A ILM Peeling	Group B ILM inverted flap	*p* value
Number	30	30	–
Male: Female	17:13	11:19	0.121^a^
Mean Age	61.17 ± 7.42 years (46.00–71.00 years)	59.37 ± 6.71 years (41.00–70.00 years)	0.328^d^
Mean Minimum Diameter	759.97 ± 85.01 μm (638.00–947.00 μm) 95% CI (728.22–791.71 μm)	803.33 ± 120.65 μm (603.00–1007.00 μm) 95% CI (758.28–848.38 μm)	0.113^d^
Mean Base Diameter	1304.50 ± 191.59 μm (873.00–1712.00 μm) 95% CI (1232.96–1376.04 μm)	1395.17 μm ± 240.56 (1005.00–1968.00 μm) 95%CI (1305.34–1484.99 μm)	0.112^d^
Mean Baseline visual acuity	logMAR 0.79 ± 0.24 (Snellen equivalent, 20/123) 95% CI (logMAR 0.70-logMAR 0.89)	logMAR 0.75 ± 0.22 (Snellen equivalent, 20/112) 95%CI (logMAR 0.66-logMAR 0.83)	0.471^c^

*ILM* Internal limiting membrane, ^a^Chi-square test, ^c^Mann-Whitney U test, ^d^independent t-test

**Fig. 1:**
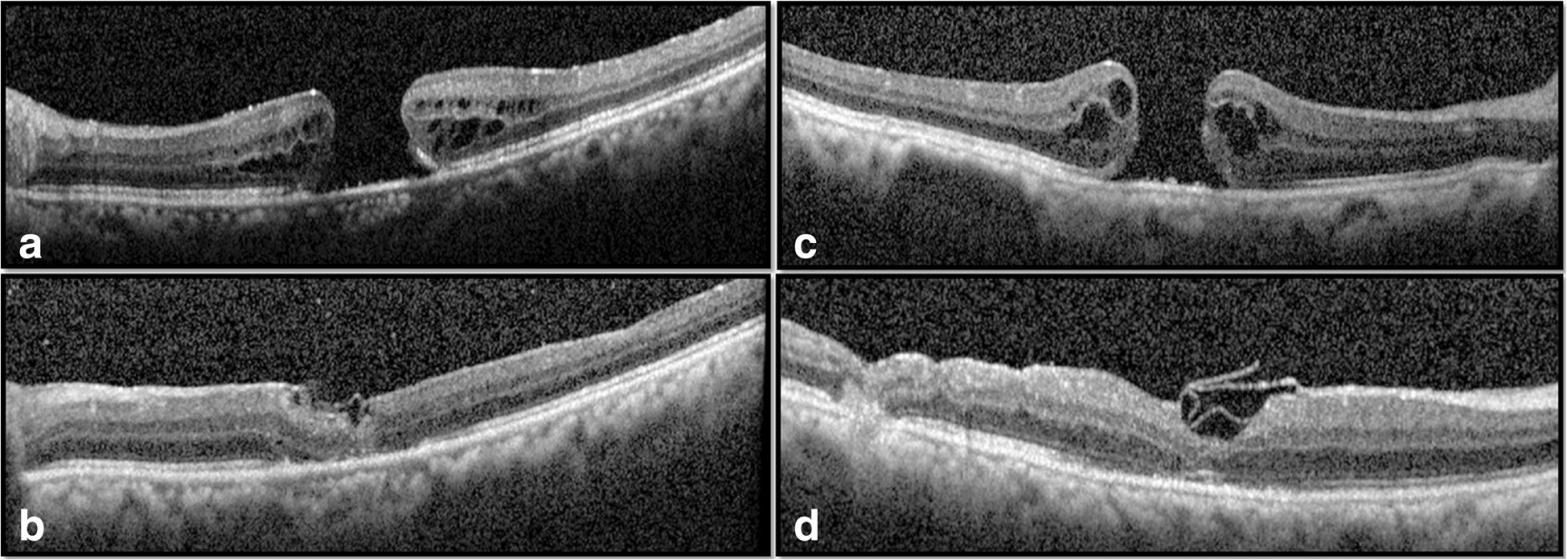
Pre-operative (**a**, **c**) and 6 months post-operative (**b**, **d**) images of two patients who underwent surgery with inverted Internal limiting membrane flap technique and achieved anatomical success

Anatomical closure was achieved in 76.7% (*n* = 23/30) and 90% (*n* = 27/30) eyes in Group A and B respectively (*p* = 0.166). Type 1 closure was achieved in 70.0% (*n* = 21/30) and 90% (n = 27/30) eyes in Group A and B respectively (Fig. 1). A two-line improvement was seen in 43.3% (*n* = 13/30) and 40.0% (*n* = 12/30) eyes in Group A and B respectively (*p* = 0.793). Mean BCVA at post-operative 1-month in Group A and Group B was logMAR 0.68 ± 0.25 (Snellen equivalent 20/96) and logMAR 0.54 ± 0.19 (Snellen equivalent 20/69) (*p* = 0.016) respectively. Mean BCVA at post-operative 6-month in Group A and B was logMAR 0.65 ± 0.25 (Snellen equivalent 20/89) and logMAR 0.53 ± 0.20 (Snellen equivalent 20/68) respectively (*p* = 0.060). The mean improvement in BCVA was 1.4 and 2.1 lines in groups A and B respectively (*p* = 0.353) (Table 2).

**Table 2:** Anatomical and functional outcome in both the groups

	Group A ILM Peeling	Group B ILM inverted flap	*p* value
Anatomical closure	76.7% (*n* = 23/30)	90.0% (*n* = 27/30)	0.166^a^
Anatomical success i.e. Type 1 closure	70.0% (*n* = 21/30)	90.0% (*n* = 27/30)	0.125^b^
Type 2 closure	6.7% (*n* = 2/30)	0	
No closure	23.3% (*n* = 7/30)	10.0% (*n* = 3/30)	
1-line improvement	46.7% (*n* = 14/30)	53.3% (*n* = 16/30)	0.606^a^
2-line improvement	43.3% (*n* = 13/30)	40.0% (*n* = 12/30)	0.793^a^
Mean BCVA at 1 month	logMAR 0.68 ± 0.25 (Snellen equivalent, 20/96).	logMAR 0.54 ± 0.19 (Snellen equivalent, 20/69)	*0.016* ^c^
Mean BCVA at 6 months	logMAR 0.65 ± 0.25 (Snellen equivalent, 20/89)	logMAR 0.53 ± 0.20 (Snellen equivalent, 20/68)	*0.060* ^c^
Mean improvement in BCVA	1.4 lines	2.1 lines	0.353^d^
BCVA≥20/60	26.7% (*n* = 8/30)	23.3% (n = 7/30)	0.766^a^

*ILM* Internal limiting membrane, *BCVA* Best-corrected visual acuity, ^a^Chi-square test, ^b^Fisher’s exact test, ^c^Mann-Whitney U test, ^d^independent t-test, in italics - statistically significant value

The three holes that did not close in the IFT group had a MD of 906 μm, 986 μm and 1007 μm. All the holes in IFT group with MD ≤ 900 μm achieved a Type 1 closure. On the contrary, four out of the seven failed surgery in ILMP group had MD < 700 μm. Anatomical closure rate 66.7% (*n* = 6/9) and 50% (*n* = 3/6) was achieved in FTMH with MD > 850 μm in IFT and ILMP group respectively. The data is available as Additional files 1 and 2.

## Discussion

Internal limiting membrane peeling relieves the tractional forces responsible for causing the hole by removing the template upon which glial tissue proliferates as well as triggers reparative gliosis by injuring the muller cells, which constitute the framework of ILM [28–32]. However, large neural defects are difficult to bridge by the glial tissue. Hence, large macular holes have a propensity to remain open or close in a Type 2 manner [6, 10, 13, 33]. Chhablani et al.*..* concluded that probability of Type1 closure with ILM peeling was 100% only if the MD of the hole was less than 300 μm [10].

In our study, a trend towards a higher anatomical success rate and a better functional outcome was noticed with inverted ILM flap technique. However, this difference did not reach statistical significance. The trend can be explained by the fact that the IFT provides a smooth and gap-free natural scaffold for the migration of glial cells and photoreceptors towards the fovea [14, 34]. Shiode et al experimentally proved that the neurotrophic and growth factors retained on the surface of the ILM flap enhanced the proliferation and migration of the muller cells. The migrating muller cells secrete neurotrophic factors and growth factors that may promote the survival of retinal neurons and photoreceptor cells. Some markers of cell proliferation like Ki-67 were also found in contact with the inverted ILM flap [35]. The technique has even been found to be superior in achieving anatomical success in case of retinal detachment associated with FTMH [36].

There have been few studies comparing the anatomical and functional outcome of IFT with conventional ILMP in case of large macular hole. However, there is no conclusive evidence suggesting the superiority of the novel technique. There are few studies which suggest that IFT is better than conventional ILMP. Michalewska et al.*.* performed a prospective trial including 50 eyes in each group. They found that anatomical closure rate was 98% in IFT group (mean MD-759 μm) and 88% in ILMP group (mean MD-698 μm) [14]. Type 1 anatomical closure rates in the IFT and ILMP groups were 96 and 69% respectively. The post-operative BCVA was significantly higher in the IFT group. Similarly, Manasa et al. did a prospective trial including 50 eyes in each group (mean MD around 650 μm in each group) [20]. They found that Type 1 closure rate was significantly better in the IFT group (62.8%) than ILMP (33.3%). Also, the functional outcome was significantly better in the IFT group. Rizzo et al. (mean MD not mentioned) in their retrospective analysis of 620 eyes, showed that both the anatomical and the functional outcome was statistically better in the IFT group (95.6%) than the ILMP group (78.6%) [21].

Other studies have found no significant difference between the two techniques. Yamashita et al. retrospectively analyzed the outcome in 165 eyes with large FTMH [22]. They found that the anatomical closure rate in the ILMP group was 95.2, 86 and 69.2% in holes with MD ≤550 μm, > 550 μm and > 700 μm respectively. On the contrary, the anatomical closure rate in the IFT group was 100% irrespective of the macular hole size. However, there was no statistically significant difference in either the anatomical or the functional outcome between the two groups. Similarly, Narayanan et al.*.* in their retrospective analysis of 36 eyes (mean MD around 550 μm in each group), found no statistically significant difference in either the anatomical or the functional outcome between the two groups [23]. Their results showed 88.9% closure rate in IFT group and 77.8% in ILM peeling group. Velez-Montoya et al performed a prospective trial with 12 patients in each group (mean MD around 600 μm in each group) [24]. They found that there was no statistically significant difference in the anatomical success rates between the two groups (91.7% in both groups). However, the functional outcome was significantly better in the IFT group.

The anatomical success rates in our study were similar to that reported in the literature. Our study showed that IFT showed a trend towards better anatomical and visual outcome in case of large macular holes. However, this difference did not reach statistically significance. In spite of not reaching clinical significance, our results show that holes with MD > 850 μm have a higher probability of closing with inverted ILM flap.

## Conclusions

The main limitation of our study was the small sample size. However, as large macular hole is an uncommon condition, it is difficult to take a large sample size operated by a single surgeon in a limited time period. Although the new technique has shown significantly better results for MH > 400 μm, it seems to be only marginally better for very large holes, especially in case of functional outcome. Larger comparative studies need to be performed to conclusively demonstrate any significant benefit of the inverted ILM flap technique.

## Additional files

Additional file 1: ᅟ(DOCX 60 kb)

Additional file 2: ᅟ(DOCX 13 kb)

## Abbreviations

BCVA: Best-corrected visual acuity
DDMS: Diamond-dusted membrane scraper
FAE: Fluid-air exchanged
FTMH: Full-Thickness macular holes
HBBG: Heavy Brilliant Blue G dye
ILM: Internal limiting membrane
IOP: Intraocular pressure
IRB: Institutional review board
MD: Minimum diameter
NSR: Neurosensory retina
RPE: Retinal pigment epithelium
SD-OCT: Spectralis Spectral-Domain Optical Coherence Tomography
SF_6_: Sulphur hexafluoride
