# Supplementary material for: Comparative study of inverted internal limiting membrane (ILM) flap and ILM peeling technique in large macular holes: a randomized-control trial
Source: BMC Ophthalmol. 2018 Jul 20;18:177. doi: 10.1186/s12886-018-0826-y (PMC6054750; doi:10.1186/s12886-018-0826-y)
Supplement: Supplementary file 1 — ᅟ(DOCX 60 kb) [file 12886_2018_826_MOESM1_ESM.docx]

**Title Page**

- **Manuscript type:** Orginal article
- **Title:** Comparative study of inverted Internal Limiting Membrane (ILM) flap and ILM peeling technique in large macular holes: A Randomized-control trial
- **Names of the authors:**

1. Dr Naresh Babu Kannan; [cauveryeye@gmail.com](mailto:cauveryeye@gmail.com)
2. Dr Piyush Kohli; [kohli119@gmail.com](mailto:kohli119@gmail.com)
3. Dr Haemoglobin Parida; [drhaemoglobinssjs@gmail.com](mailto:drhaemoglobinssjs@gmail.com)
4. OO Adenuga; [korexmed@yahoo.com](mailto:korexmed@yahoo.com)
5. Dr Kim Ramasamy; [kim@aravind.org](mailto:kim@aravind.org)

- **Affiliation of authors:**  Department of Vitreo-retinal services, Aravind Eye Hospital and Post graduate Institute of Ophthalmology, Madurai, Tamil Nadu, India
- **Corresponding author**

Dr. Kannan NB

Senior medical consultant,

Department of Vitreo-retinal services, Aravind Eye Hospital and Post graduate Institute of Ophthalmology, Madurai, Tamil Nadu, India

[cauveryeye@gmail.com](mailto:cauveryeye@gmail.com)

- No conflict of interests

**Abstract**

**Aim**

The anatomical success rate of macular hole surgery ranges around 93-98%. However, the prognosis of large macular holes is generally poor. The study was conducted to compare the anatomical and visual outcomes of Internal Limiting Membrane (ILM) peeling vis-a-vis inverted ILM flap for the treatment of idiopathic large Full-Thickness Macular Holes (FTMH).

**Material and methods**

This was a prospective randomized control trial. The study included patients with idiopathic FTMH, with a minimum diameter ranging from 600-1500μm. The patients were randomized into Group A (ILM peeling) and Group B (inverted ILM flap). The main outcome measures were anatomical and visual outcome at the end of 6 months. Anatomical success was defined as flattening of macular hole with resolution of the subretinal cuff of fluid and neurosensory retina completely covering the fovea.

**Results**

There were 30 patients in each group. The mean minimum diameters in Group A and B were 759.97±85.01μm and 803.33±120.65μm respectively (p=0.113). The mean base diameter in group A and B was 1304.50±191.59μm and 1395.17±240.56μm respectively (p=0.112). The anatomical success rates achieved in Group A and B were 70.0% and 90.0% respectively (p=0.125). The mean best-corrected visual acuity (BCVA) after 6 months was logMAR 0.65±0.25 (Snellen equivalent, 20/89) in Group A and logMAR 0.53±0.20 (Snellen equivalent, 20/68) in Group B (p=0.060). The mean improvement in BCVA was 1.4 lines and 2.1 lines in groups A and B respectively (p=0.353). BCVA≥20/60 was achieved by 13.3% and 20.0% in group A and B respectively (p=0.766).

**Conclusion**

The anatomical and functional outcome of Inverted ILM flap technique in large FTMH is statistically similar to that seen in conventional ILM peeling.

**Trial Registration (retrospectively registered):**  Clinical Trials Registry – India (Indian Medical Research) CTRI/2017/11/010474.

**Keywords**

1. 600 microns

2. Inverted ILM flap

3. Large macular hole

4. Muller cells

5. Type 1 closure

**Introduction**

Vitrectomy is the gold standard for the treatment of macular holes. The anatomical success rate of macular hole surgery is 93-98%.^1-4^ However, the anatomical success rate of large macular holes is as low as 40 to 80%.^5-10^ Poor prognosis demoralizes most surgeons from operating upon such patients.^11-13^

Michalewska *et al*, first described a novel technique of inverted internal limiting membrane (ILM) flap for the treatment of large macular holes.^14^ They found that their technique achieved better anatomical and visual outcomes compared to conventional ILM peeling (ILMP). In last couple of years, a number of studies have suggested that inverted ILM flap technique (IFT) may be better for the treatment of large macular holes.^14-24^ A systemic review and single-arm meta-analysis showed that the anatomical closure and visual improvement rates after IFT for FTMH with minimum diameter (MD) >400μm were 95% and 75% respectively.^19^ But most of these studies were either retrospective in nature or lacked a control arm and included macular hole with MD<600μm.

We performed a randomized control trial to compare the anatomical and visual outcome of inverted ILM flap technique (IFT) vis-à-vis conventional ILM peeling (ILMP) in idiopathic large macular holes with MD>600μm.

**Materials and Methods**

This was a prospective randomized control study done at Aravind Eye Hospital, Madurai, India, after obtaining approval from Institutional Review Board (IRB) of Aravind Medical Research Foundation (Registration No. ECR/182/Inst/TN/2013 dated 20.04.2013). This study adheres to the tenets of Declaration of Helsinki. The nature and aim of the study was explained to the patients, and a written consent for participation was taken from each patient before the surgery. Patients with idiopathic full thickness macular hole (FTMH) with a MD>600μm were included. Patients with MD>1500μm, traumatic macular holes, myopic macular holes, presence of co-existing ocular pathologies affecting vision and patients refusing randomization were excluded from the study.

The presenting best-corrected visual acuity (BCVA) and intraocular pressure (IOP) were recorded. The Snellen visual acuity was converted into a logarithm of the minimum angle of resolution i.e. logMAR for statistical analysis. FTMH parameters and indices were gauged with Heidelberg Spectralis Spectral-Domain Optical Coherence Tomography (SD-OCT) (Heidelberg Engineering, Inc., Heidelberg, Germany) using high definition 5-line raster scans and 3-dimensional 512×128 macular cube scans passing through the fovea, before and after the surgery.^6,7,9^ All the surgeries in both the groups were performed by a single surgeon (Dr NB) with more than 15 years of post-fellowship experience of performing high volume vitreoretinal surgeries. All the patients were randomized into two groups. System-generated random number were used to recruit the patients into two groups. The patients in group A underwent 25G Pars Plana Vitrectomy (PPV) with ILMP while the patients in group B underwent 25G PPV with IFT.

**Surgical technique**

In both groups, phacoemulsification with implantation of intraocular lens was followed by core vitrectomy and induction of posterior vitreous detachment. ILM was then stained with 0.05% solution of Heavy Brilliant Blue G dye (HBBG), prepared by mixing Brilliant Blue G dye (Ocublue plus, Aurolab, India) with 10% dextrose in 1:2 proportions. HBBG was injected slowly under balance salt solution (BSS).^25^ The stained ILM was pinched with a 25G end gripping forceps (Grieshaber Asymmetrical Forceps, DSP, Alcon, Fort Worth, Texas, USA) and peeled off in a circular fashion for approximately 2-disc diameters around the hole.

In the ILM peeling group, the ILM was discarded. In the inverted ILM flap group, the margins of the ILM were left attached to the edges of the hole. The margins were later trimmed with the vitrectomy cutter. Only adequate amount of ILM required to tuck into the hole was retained. Fluid-air exchange (FAE) was done and the ILM flap was tucked into hole with Tano diamond-dusted membrane scraper (DDMS; Synergetics, Inc., O'Fallon, MO, USA).

In both groups, FAE was done multiple times to ensure complete fluid removal. The superonasal and superotemporal cannulae were then removed and the conjunctiva was repositioned to cover the sclerotomy sites. Two mL pure SF_6_ was injected with a 30-gauge needle, while the air-infusion line was used for venting. After the syringe was flushed, the infusion line was clamped and the digital tension of the globe was assessed. The infusion cannula was then removed and the inferotemporal sclerotomy sealed.^26^ Post-operative prone positioning was recommended for first 48 hours.

**Post-operative Evaluation**

Post-operative visits were scheduled at day1, 2weeks, 1month and 6months. Frequent follow-ups were scheduled, in case of any complication. At each follow-up visit BCVA, IOP and SD-OCT were recorded. The main outcome measures were anatomical and visual outcome at the end of 6 months. Anatomical closure was defined as the flattening of the hole with resolution of subretinal cuff of fluid. Anatomical success was defined as Type 1 anatomical closure i.e. flattening of macular hole with resolution of subretinal cuff of fluid and neurosensory retina (NSR) completely covering the fovea.^27^ Type 2 anatomical closure, i.e. when the whole rim of the NSR around the macular hole was attached to the underlying retinal pigment epithelium (RPE) but NSR was absent above the fovea, was also considered anatomical failure.

**Statistical analysis**

Statistical analysis was performed by using statistical software STATA 14.1, (Texas, USA). Continuous variables were expressed as mean (±standard deviation) or median (range) and categorical variables were expressed as percentages. Chi-square test/ Fisher’s exact test was used to assess the association of categorical variables. Student’s t-test/ Mann-Whitney U test was used to find out the significant difference of continuous variables between the two study groups. Wilcoxon sign rank test was used to find out the difference between pre- and post-operative visual acuity. P-value less than 0.05 considered as statistically significant.

**Sample size calculation**

The type 1 closure rate obtained by Michalewska *et al*, i.e 69% in ILMP group and 96% in IFT group, was used as reference.^14^ By keeping the power of the study as 80% and the confidence interval as 95%, a sample of 60 subjects (30 in each arm) was calculated. The following formula was used:


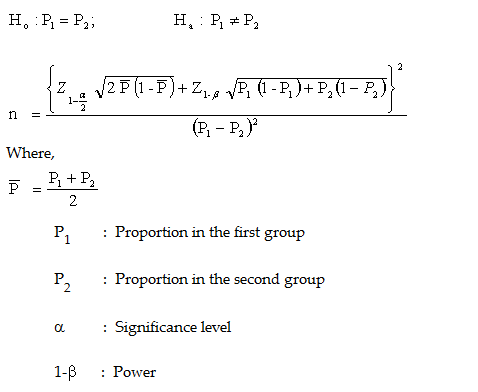


**Results**

The study included 30 patients in each of the two groups. The mean MD in group A (ILMP) and group B (IFT) was 759.97±85.01μm and 803.33±120.65μm respectively (p=0.113). The mean base diameter in group A and B was 1304.50±191.59μm and 1395.17±240.56μm respectively (p=0.112). The mean BCVA in group A and B was logMAR 0.79±0.24 (Snellen equivalent 20/123) and logMAR 0.75±0.22 (Snellen equivalent 20/112) respectively (p=0.471) (Table 1).

Anatomical closure was achieved in 76.7% (n=23/30) and 90% (n=27/30) eyes in Group A and B respectively (p=0.166). Type 1 closure was achieved in 70.0% (n=21/30) and 90% (n=27/30) eyes in Group A and B respectively (Figure 1). A two-line improvement was seen in 43.3% (n=13/30) and 40.0% (n=12/30) eyes in Group A and B respectively (p=0.793). Mean BCVA at post-operative 1-month in Group A and Group B was logMAR 0.68±0.25 (Snellen equivalent 20/96) and logMAR 0.54±0.19 (Snellen equivalent 20/69) (p=0.016) respectively. Mean BCVA at post-operative 6-month in Group A and B was logMAR 0.65±0.25 (Snellen equivalent 20/89) and logMAR 0.53±0.20 (Snellen equivalent 20/68) respectively (p=0.060). The mean improvement in BCVA was 1.4 and 2.1 lines in groups A and B respectively (p=0.353) (Table 2).

The three holes that did not close in the IFT group had a MD of 906μm, 986μm and 1007μm. All the holes in IFT group with MD≤900μm achieved a Type 1 closure. On the contrary, four out of the seven failed surgery in ILMP group had MD<700μm. Anatomical closure rate 66.7% (n=6/9) and 50% (n=3/6) was achieved in FTMH with MD>850μm in IFT and ILMP group respectively.

**Discussion**

Internal limiting membrane peeling relieves the tractional forces responsible for causing the hole by removing the template upon which glial tissue proliferates as well as triggers reparative gliosis by injuring the muller cells, which constitute the framework of ILM.^28-32^ However, large neural defects are difficult to bridge by the glial tissue. Hence, large macular holes have a propensity to remain open or close in a Type 2 manner.^6,10,13,33^ Chhablani *et al* concluded that probability of Type1 closure with ILM peeling was 100% only if the MD of the hole was less than 300μm.^10^

In our study, a trend towards a higher anatomical success rate and a better functional outcome was noticed with inverted ILM flap technique. However, this difference did not reach statistical significance. The trend can be explained by the fact that the IFT provides a smooth and gap-free natural scaffold for the migration of glial cells and photoreceptors towards the fovea.^14,34^ Shiode *et al* experimentally proved that the neurotrophic and growth factors retained on the surface of the ILM flap enhanced the proliferation and migration of the muller cells. The migrating muller cells secrete neurotrophic factors and growth factors that may promote the survival of retinal neurons and photoreceptor cells. Some markers of cell proliferation like Ki-67 were also found in contact with the inverted ILM flap.^35^ The technique has even been found to be superior in achieving anatomical success in case of retinal detachment associated with FTMH.^36^

There have been few studies comparing the anatomical and functional outcome of IFT with conventional ILMP in case of large macular hole. However, there is no conclusive evidence suggesting the superiority of the novel technique. There are few studies which suggest that IFT is better than conventional ILMP. Michalewska *et al* performed a prospective trial including 50 eyes in each group. They found that anatomical closure rate was 98% in IFT group (mean MD-759μm) and 88% in ILMP group (mean MD-698μm).^14^ Type 1 anatomical closure rates in the IFT and ILMP groups were 96% and 69% respectively. The post-operative BCVA was significantly higher in the IFT group. Similarly, Manasa *et al* did a prospective trial including 50 eyes in each group (mean MD around 650μm in each group).^20^ They found that Type 1 closure rate was significantly better in the IFT group (62.8%) than ILMP (33.3%). Also, the functional outcome was significantly better in the IFT group. Rizzo *et al* (mean MD not mentioned) in their retrospective analysis of 620 eyes, showed that both the anatomical and the functional outcome was statistically better in the IFT group (95.6%) than the ILMP group (78.6%).^21^

Other studies have found no significant difference between the two techniques. Yamashita *et al* retrospectively analyzed the outcome in 165 eyes with large FTMH.^22^ They found that the anatomical closure rate in the ILMP group was 95.2%, 86% and 69.2% in holes with MD ≤550μm, >550μm and >700μm respectively. On the contrary, the anatomical closure rate in the IFT group was 100% irrespective of the macular hole size. However, there was no statistically significant difference in either the anatomical or the functional outcome between the two groups. Similarly, Narayanan *et al* in their retrospective analysis of 36 eyes (mean MD around 550μm in each group), found no statistically significant difference in either the anatomical or the functional outcome between the two groups.^23^ Their results showed 88.9% closure rate in IFT group and 77.8% in ILM peeling group. Velez‑Montoya *et al* performed a prospective trial with 12 patients in each group (mean MD around 600μm in each group).^24^ They found that there was no statistically significant difference in the anatomical success rates between the two groups (91.7% in both groups). However, the functional outcome was significantly better in the IFT group.

The anatomical success rates in our study were similar to that reported in the literature. Our study showed that IFT showed a trend towards better anatomical and visual outcome in case of large macular holes. However, this difference did not reach statistically significance. In spite of not reaching clinical significance, our results show that holes with MD>850μm have a higher probability of closing with inverted ILM flap.

The main limitation of our study was the small sample size. However, as large macular hole is an uncommon condition, it is difficult to take a large sample size operated by a single surgeon in a limited time period. Although the new technique has shown significantly better results for MH>400μm, it seems to be only marginally better for very large holes, especially in case of functional outcome. Larger comparative studies need to be performed to conclusively demonstrate any significant benefit of the inverted ILM flap technique.

**List of abbreviations**

ILM - Internal Limiting Membrane

FTMH - Full-Thickness Macular Holes

HBBG- Heavy Brilliant Blue G dye

SF_6_-Sulphur hexafluoride

IOP - Intraocular pressure

BCVA - Best-Corrected Visual Acuity

IRB - Institutional Review Board

MD - Minimum diameter

SD-OCT - Spectralis Spectral-Domain Optical Coherence Tomography

FAE - Fluid-air exchanged

DDMS- Diamond-dusted membrane scraper

NSR - Neurosensory Retina

RPE - Retinal pigment epithelium

**Declarations**

- **Ethics approval and written consent to participate** - was taken from the Institutional review board of Aravind Medical Research Foundation (Registration No. ECR/182/Inst/TN/2013 dated 20.04.2013). The trial was “retrospectively registered” with Clinical Trials Registry – India (Indian Medical Research). A written consent for participation was taken from each patient before the surgery
- **Consent for publication** was taken in writing from all the patients before surgery. All the patients in the study consented for their individual data to be published.
- **Availability of data and material:** All data generated or analyzed during this study are included in this published article [and its supplementary information files]. Any additional information can be requested from the corresponding author
- **Competing interests** – Not applicable
- **Funding** - Not applicable
- **Authors' contributions**

1. Dr NBK - Research Design, Data Interpretation, Manuscript Preparation
2. Dr PK - Data Interpretation, Manuscript Preparation
3. Dr HP - Research Design, Data Acquisition, Data Interpretation, Manuscript Preparation
4. Dr OOA - Data Interpretation, Manuscript Preparation
5. Dr KR - Research Design, Data Interpretation

- **Acknowledgements** - Not applicable

**References**

1. Rahimy E, MCCannel CA. Impact of internal limiting membrane peeling on macular hole reopening. A systematic review and meta-analysis. *Retina.* 2016;36:679-87. doi:[10.1097/IAE.0000000000000782](https://doi.org/10.1097/IAE.0000000000000782)

# 2. Lai MM, Williams GA. Anatomical and visual outcomes of idiopathic macular hole surgery with internal limiting membrane removal using low-concentration indocyanine green. *Retina.* 2007;27:477-82. doi:[10.1097/01.iae.0000247166.11120.21](https://doi.org/10.1097/01.iae.0000247166.11120.21)

3. Park DW, Sipperley JO, Sneed SR, Dugel PU, Jacobsen J. Macular hole surgery with internal-limiting membrane peeling and intravitreous air. *Ophthalmology.* 1999;106:1392-98. doi:[10.1016/S0161-6420(99)00730-7](https://doi.org/10.1016/S0161-6420(99)00730-7)

4. Wolf S, Reichel MB, Wiedemann P, Schnurrbusch UE. Clinical findings in macular hole surgery with indocyanine greenassisted peeling of the internal limiting membrane. *Graefes Arch Clin Exp Ophthalmol.* 2003;241:589–592. doi:[10.1007/s00417-003-0673-1](https://doi.org/10.1007/s00417-003-0673-1)

5. Ip MS, Baker BJ, Duker JS, Reichel E, Baumal CR, Gangnon R, *et al*. Anatomical outcomes of surgery for idiopathic macular hole as determined by optical coherence tomography. *Arch Ophthalmol.* 2002;120:29-35.

6. Ullrich S, Haritoglou C, Gass C,  Schaumberger M, Ulbig MW, Kampik A. Macular hole size as a prognostic factor in macular hole surgery. *Br J Ophthalmol.* 2002;86:390-93.

7. Kusuhara S, Teraoka MF, Fujii S, Nakanishi Y, Tamura Y, Nagai A, *et al.* Prediction of postoperative visual outcome based on hole configuration by optical coherence tomography in eyes with idiopathic macular holes. *Am J Ophthalmol.* 2004;138:709–16. doi:[10.1016/j.ajo.2004.04.063](https://doi.org/10.1016/j.ajo.2004.04.063)

8. Haritoglou C, Neubauer AS, Reiniger IW, Priglinger SG, Gass CA, Kampik A. Long-term functional outcome of macular hole surgery correlated to optical coherence tomography measurements. *Clin Exp Ophthalmol.* 2007;35:208–13. doi:[10.1111/j.1442-9071.2006.01445.x](https://doi.org/10.1111/j.1442-9071.2006.01445.x)

9. Ruiz-Moreno JM, Staicu C, Piñero DP, Montero J, Lugo F, Amat P. Optical coherence tomography predictive factors for macular hole surgery outcome. *Br J Ophthalmol.* 2008;92: 640-44. doi: [10.1136/bjo.2007.136176](https://doi.org/10.1136/bjo.2007.136176)

10. Chhablani J, Khodani M, Hussein A, Bondalapati S, Rao HB, Narayanan R*.* Role of macular hole angle in macular hole closure. *Br J Ophthalmol.* 2015;99:1634-38. doi: [10.1136/bjophthalmol-2015-307014](https://doi.org/10.1136/bjophthalmol-2015-307014)

11. Scott RA, Ezra E, West JF, Gregor ZJ. Visual and anatomical results of surgery for long standing macular holes. *Br J Ophthalmol.* 2000;84:150-3.

12. Stec LA, Ross RD, Williams GA, Gregor ZJ. Vitrectomy for chronic macular holes. *Retina.* 2004;24:341-7.

# 13. Shukla SY, Afshar AR, Kiernan DF, Hariprasad SM. Outcomes of chronic macular hole surgical repair. *Indian J Ophthalmol.* 2014;62:795-8. doi: [10.4103/0301-4738.138302](https://doi.org/10.4103/0301-4738.138302)

14. Michalewska Z, Michalewski J, Adelman RA, Nawrocki J. Inverted internal limiting membrane flap technique for large macular holes. *Ophthalmology.* 2010;117:2018-25. doi: [10.1016/j.ophtha.2010.02.011](https://doi.org/10.1016/j.ophtha.2010.02.011)

# 15. Mahalingam P, Sambhav K. Surgical outcomes of inverted internal limiting membrane flap technique for large macular hole. *Indian J Ophthalmol.* 2013;61:601-3. doi: [10.4103/0301-4738.121090](https://doi.org/10.4103/0301-4738.121090)

16. Shanmugam MP, Ramanjulu R, Kumar M, Rodrigues G, Reddy S, Mishra D. Inverted ILM peeling for idiopathic and other etiology macular holes. *Indian J Ophthalmol.* 2014;62:898-9. doi:  [10.4103/0301-4738.141077](https://doi.org/10.4103/0301-4738.141077)

17. Khodani M, Bansal P, Narayanan R, Chhablani J. Inverted internal limiting membrane flap technique for very large macular hole. *Int J Ophthalmol.* 2016; 9:1230-2. doi: [10.18240/ijo.2016.08.22](https://doi.org/10.18240/ijo.2016.08.22)

18. Chen Z, Zhao C, Ye JJ, Sui RF. Inverted Internal Limiting Membrane flap technique for repair of large macular holes: A short-term follow-up of anatomical and functional outcomes. *Chin Med J (Engl).* 2016;129:511-17. doi: [10.4103/0366-6999.176988](https://doi.org/10.4103/0366-6999.176988)

19. Gu C, Qiu Q. Inverted internal limiting membrane flap technique for large macular holes: a systematic review and single-arm meta-analysis. *Graefes Arch Clin Exp Ophthalmol.* 2018 [Ahead of print]. doi: 10.1007/s00417-018-3956-2.

20. Manasa S, Kakkar P, Kumar A, Chandra P, Kumar V, Ravani R. Comparative evaluation of standard ILM Peel with inverted ILM flap technique in large macular Holes: A prospective, randomized study. *Ophthalmic Surg Lasers Imaging Retina.* 2018;49:236-240. doi:10.3928/23258160-20180329-04.

21. Rizzo S, Tartaro R, Barca F, Caporossi T, Bacherini D, Giansanti F. Internal limiting membrane peeling versus inverted flap technique for treatment of full-thickness macular holes: A comparative study in a large series of patients. Retina. 2017 Dec 8 [Ahead of print]. doi:10.1097/IAE.0000000000001985.

22. Yamashita T, Sakamoto T, Terasaki H, Iwasaki M, Ogushi Y, Okamoto F, *et al*. Best surgical technique and outcomes for large macular holes: retrospective multicentre study in Japan. *Acta Ophthalmol.* 2018 Apr 19 [Ahead of print]. doi: 10.1111/aos.13795.

23. Narayanan R, Singh SR, Taylor S, Berrocal MH, Chhablani J, Tyagi M, *et al*. Surgical outcomes after inverted internal limiting membrane flap versus conventional peeling for very large macular holes. *Retina.* 2018 Apr 23 [Ahead of print]. doi:10.1097/IAE.0000000000002186.

24. Velez-Montoya R, Ramirez-Estudillo JA, Sjoholm-Gomez de Liano C, Bejar-Cornejo F, Sanchez-Ramos J, Guerrero-Naranjo JL, *et al.* Inverted ILM flap, free ILM flap and conventional ILM peeling for large macular holes. *Int J Retina Vitreous.* 2018;4:8. doi:10.1186/s40942-018-0111-5.

25. Shukla D, Kalliath J, Patwardhan A, Kannan NB, Thayyil SB. A preliminary study of Heavy Brilliant Blue G for internal limiting membrane staining in macular hole surgery. *Indian J Ophthalmol.* 2012;60:531-4. doi: [10.4103/0301-4738.103786](https://doi.org/10.4103/0301-4738.103786)

26. Kannan NB, Adenuga OO, Kumar K, Ramasamy K. Outcome of 2 cc pure sulfur hexafluoride gas tamponade for macular hole surgery. *BMC Ophthalmol.* 2016;16:73. doi: [10.1186/s12886-016-0254-9](https://doi.org/10.1186/s12886-016-0254-9)

27. Landolfi M, Zarbin MA, Bhagat N. Macular holes. Ophthalmol Clin North Am. 2002;15:565-72.

28. Smiddy WE, Feuer W, Cordahi G. Internal limiting membrane peeling in macular hole surgery. *Ophthalmology.* 2001;108:1471-76.

29. Gupta D. Face-down posturing after macular hole surgery. A Review. *Retina.* 2009; 29:430-43. doi: [10.1097/IAE.0b013e3181a0bd01](https://doi.org/10.1097/IAE.0b013e3181a0bd01)

30. Funata M, Wendel RT, de la Cruz Z, Green WR. Clinicopathologic study of bilateral macular holes treated with pars plana vitrectomy and gas tamponade. *Retina.* 1992;12:289 –98.

31. Madreperla SA, Geiger GL, Funata M, de la Cruz Z, Green WR. Clinicopathologic correlation of a macular hole treated by cortical vitreous peeling and gas tamponade. *Ophthalmology.* 1994;101:682– 6.

32. Rosa RH Jr, Glaser BM, de la Cruz Z, Green WR. Clinicopathologic correlation of an untreated macular hole and a macular hole treated by vitrectomy, transforming growth factor-b2, and gas tamponade. *Am J Ophthalmol.* 1996;122:853- 63.

# 33. Kang SW, Ahn K, Ham DI. Types of macular hole closure and their clinical implications. *Br J Ophthalmol.* 2003;87:1015-19.

34. Michalewska Z, Michalewski J, Dulczewska-Cichecka K, Adelman RA, Nawrocki J. Temporal inverted internal limiting membrane flap technique versus classic inverted internal limiting membrane flap technique: A Comparative Study. *Retina* 2015; 35: 1844-50. doi: [10.1097/IAE.0000000000000555](https://doi.org/10.1097/IAE.0000000000000555)

35. Shiode Y, Morizane Y, Matoba R, Hirano M, Doi S, Toshima S, *et al*. The role of inverted internal limiting membrane flap in macular hole closure. *Invest Ophthalmol Vis Sci.* 2017;58:4847-55. doi: 10.1167/iovs.17-21756.

36. Yuan J, Zhang LL, Lu YJ, Han MY, Yu AH, Cai XJ. Vitrectomy with internal limiting membrane peeling versus inverted internal limiting membrane flap technique for macular hole-induced retinal detachment: a systematic review of literature and meta-analysis. *BMC Ophthalmol.* 2017;17:219. doi:10.1186/s12886-017-0619-8.

**Legends**

Figure 1: Pre-operative (a,c) and 6 months post-operative (b,d) images of two patients who underwent surgery with inverted Internal limiting membrane flap technique and achieved anatomical success.
