# Supplementary material for: Comparative study of inverted internal limiting membrane (ILM) flap and ILM peeling technique in large macular holes: a randomized-control trial
Source: BMC Ophthalmol. 2018 Jul 20;18:177. doi: 10.1186/s12886-018-0826-y (PMC6054750; doi:10.1186/s12886-018-0826-y)
Supplement: Supplementary file 2 — ᅟ(DOCX 13 kb) [file 12886_2018_826_MOESM2_ESM.docx]

File name: 3.6.18

Title of data: raw data for comparison of inverted Internal Limiting Membrane (ILM) flap with ILM peeling technique in large macular holes

Description of data:

1. Age is in years
2. Sex-M is male & F is female
3. Pre-VA (L) means Pre-operative visual acuity in logMAR
4. Min D mean minimum diameter of the macular hole
5. BASE D mean Base diameter of the macular hole
6. Result means Type of macular hole closure. 1-means Type 1 closure, 2-means Type 2 closure and Open means that the hole did not close
7. VA_1m means 1-month post-operative visual acuity in logMAR;
8. VA_6m means 6-month post-operative visual acuity in logMAR;
9. 1L imp means 1-line improvement; 0 means No, 1 means Yes
10. 2L imp means 2-line improvement; 0 means No, 1 means Yes
11. BCVA≥20/60 means Best corrected visual acuity >20/60 at 6-month post-operative visit; 0 means No, 1 means Yes
